# Age differences in functioning and contextual factors in community-dwelling stroke survivors: A national cross-sectional survey

**DOI:** 10.1371/journal.pone.0273644

**Published:** 2022-08-25

**Authors:** Steinunn A. Olafsdottir, Ingibjörg Hjaltadottir, Rose Galvin, Thora B. Hafsteinsdottir, Helga Jonsdottir, Solveig A. Arnadottir

**Affiliations:** 1 Faculty of Medicine, Department of Physical Therapy, School of Health Sciences, University of Iceland, Reykjavík, Iceland; 2 Faculty of Nursing, School of Health Sciences, University of Iceland, Reykjavík, Iceland; 3 Division of Clinical Services I, Landspitali- The National University Hospital of Iceland, Reykjavík, Iceland; 4 School of Allied Health, Ageing Research Centre, Health Research Institute, University of Limerick, Limerick, Ireland; 5 Julius Center for Health Sciences and Primary Care, University Medical Center Utrecht, Utrecht, The Netherlands; Cardiff University, UNITED KINGDOM

## Abstract

**Background:**

Our study aimed to map functioning and contextual factors among community-dwelling stroke survivors after first stroke, based on the International Classification of Functioning, Disability and Health (ICF), and to explore if these factors differ among older-old (75 years and older), younger-old (65–74 years), and young (18–65 years) stroke survivors.

**Methods:**

A cross-sectional population-based national survey among community-dwelling stroke survivors, 1–2 years after their first stroke. Potential participants were approached through hospital registries. The survey had 56.2% response rate. Participants (N = 114, 50% men), 27 to 94 years old (71.6±12.9 years), were categorized as: older-old (n = 51), younger-old (n = 34) and young (n = 29). They answered questions on health, functioning and contextual factors, the Stroke Impact Scale (SIS) and the Behavioural Regulation Exercise Questionnaire-2. Descriptive analysis was used, along with analysis of variance for continuous data and Fisher´s exact tests for categorical variables. TukeyHSD, was used for comparing possible age-group pairings.

**Results:**

The responses reflected ICF´s personal and environmental factors as well as body function, activities, and participation. Comparisons between age-groups revealed that the oldest participants reported more anxiety and depression and used more walking devices and fewer smart devices than individuals in both the younger-old and young groups. In the SIS, the oldest participants had lower scores than both younger groups in the domains of activities of daily living and mobility.

**Conclusion:**

These findings provide important information on needs and opportunities in community-based rehabilitation for first-time stroke survivors and reveal that this population has good access to smart devices which can be used in community integration. Moreover, our results support the need for analysis in subgroups of age among the heterogenous group of older individuals in this population.

## Introduction

Stroke is one of the primary causes of chronic disability in the Western world [1]. The incidence of stroke increases with age [1], and despite the fact that stroke can happen at any age, 75% of all strokes are among adults older than 65 years of age [2]. After hospitalization and/or inpatient rehabilitation, the majority of stroke survivors are discharged home where they may need appropriate community-based rehabilitation to maximize their functioning [3–5]. For effective rehabilitation interventions and community integration, it is crucial to understand the complex underlying factors that create rehabilitation needs and contribute to positive integration outcomes. Many recent studies have focused on innovative technical interventions and smart devices to use in community-based rehabilitation for stroke survivors [6] and during the ongoing Covid-19 pandemic, there has been a surge in the implementation of telerehabilitation for these clients, which includes use of smart devices [7]. Therefore, it is important to recognise the access and use smart devices in different age groups as well as the age-related differences in the recovery post-stroke among community-dwelling stroke survivors. Moreover, the theoretical framework from the World Health Organization, International Classification of Functioning, Disability and Health (ICF) [8] is useful to map and recognise various factors in surveys and to identify the opportunities for community-based rehabilitation and integration [9].

Among these underlying factors are age-related changes in physical, cognitive, personal and psychosocial function affect the health and functioning of each individual [10]. Therefore, older community-dwelling stroke survivors may be more challenged than younger ones with impairments after stroke in addition to age-related disability. In addition, older stroke survivors might be less willing to use modern telerehabilitation due to attitudes towards technology and computer anxiety [11]. Despite that, stroke survivors are often presented as one group in studies, regardless of age [3,12–17]. Given the high incidence of stroke in the older population, heterogeneity among people who have reached the age of 65 years, and the worldwide emphasis on aging in place, only a limited number of studies have attempted to gain a deeper understanding of older age on community-dwelling stroke survivors [18–20]. Some studies have used the cut-off age of 65 years to compare stroke survivors, and only revealed minor differences in functioning between the age-groups [21,22], indicating the need to improve the consideration of age in more subgroups. These studies may not have captured the important variations in functioning and contextual factors among the heterogeneous group of stroke survivors older than 65 years old.

Applying gerontological theory in stroke research may be a useful approach for older age categorization in the stroke literature. Within gerontological research, the classic definition of *old* has been 65 years, the age when individuals can retire [23] and collect social security benefits [24]. On the other hand, there has been a call for changing this definition of old to 75 years of age based on increased life expectancy, functional independence and more employment of older people [25–27]. Research on stroke may benefit from exploring how the definition of old age being 75 years of age fits the population of older stroke survivors, and whether either cut-off point (75 or 65 years) is helpful in creating meaningful older age categories among stroke survivors who are healthy enough to be community-dwelling. Based on this, the group aged 75 years and older could be categorized as old and reflect people who are expected to have substantial age-related changes in functioning and social roles; the group aged 65–74 years old could be categorized as younger-old and reflect people who are approaching older age with potential age-related changes in social roles and functioning; and those who are younger than 65 years could be categorized as young and middle-aged and reflect those who are expected to be following their career and engaged with family life.

The heterogeneous group of community-dwelling stroke survivors across a wide age-span and different disabilities needs diverse rehabilitation that is tailored to the needs of the individuals, as well as support from the community to optimize their quality of life after stroke. Therefore, it is important to gain a thorough understanding of the functioning and contextual factors and to examine further how older age affects this population. Our study aimed to: 1) map the functioning and contextual factors among community-dwelling stroke survivors one to two years after their first stroke, based on the different components of the ICF [8], and 2) to explore if functioning and contextual factors of this population differ among old (75 years and older), younger-old (65–74 years), and young and middle-aged stroke survivors (18–65 years).

## Methods

### Study design and participants

A cross-sectional population-based survey was mailed to eligible community-dwelling adult stroke survivors (individuals living in their homes but not institutions) who had been diagnosed with their first stroke one to two years earlier. Potential participants were identified through registries from the two main hospitals in Iceland, which gave the opportunity to approach the whole population diagnosed with stroke in one year. To be defined as eligible the following inclusion criteria were used: Admission to one of the two hospitals within a 12-month period (April 1^st^ 2016 –March 31^st^ 2017) with the diagnosis of stroke (ICD10 I60-I64) for the first time, and at least 18 years old when diagnosed. Exclusion criteria were: Diagnosis of dementia (ICD10 F00-F03) prior to the time of the study, living in a nursing home, not having an Icelandic national insurance number and living abroad. The STROBE standardized reporting guidelines [28] were followed to standardize the conduct and reporting of the study.

### The survey

**Fig 1:**
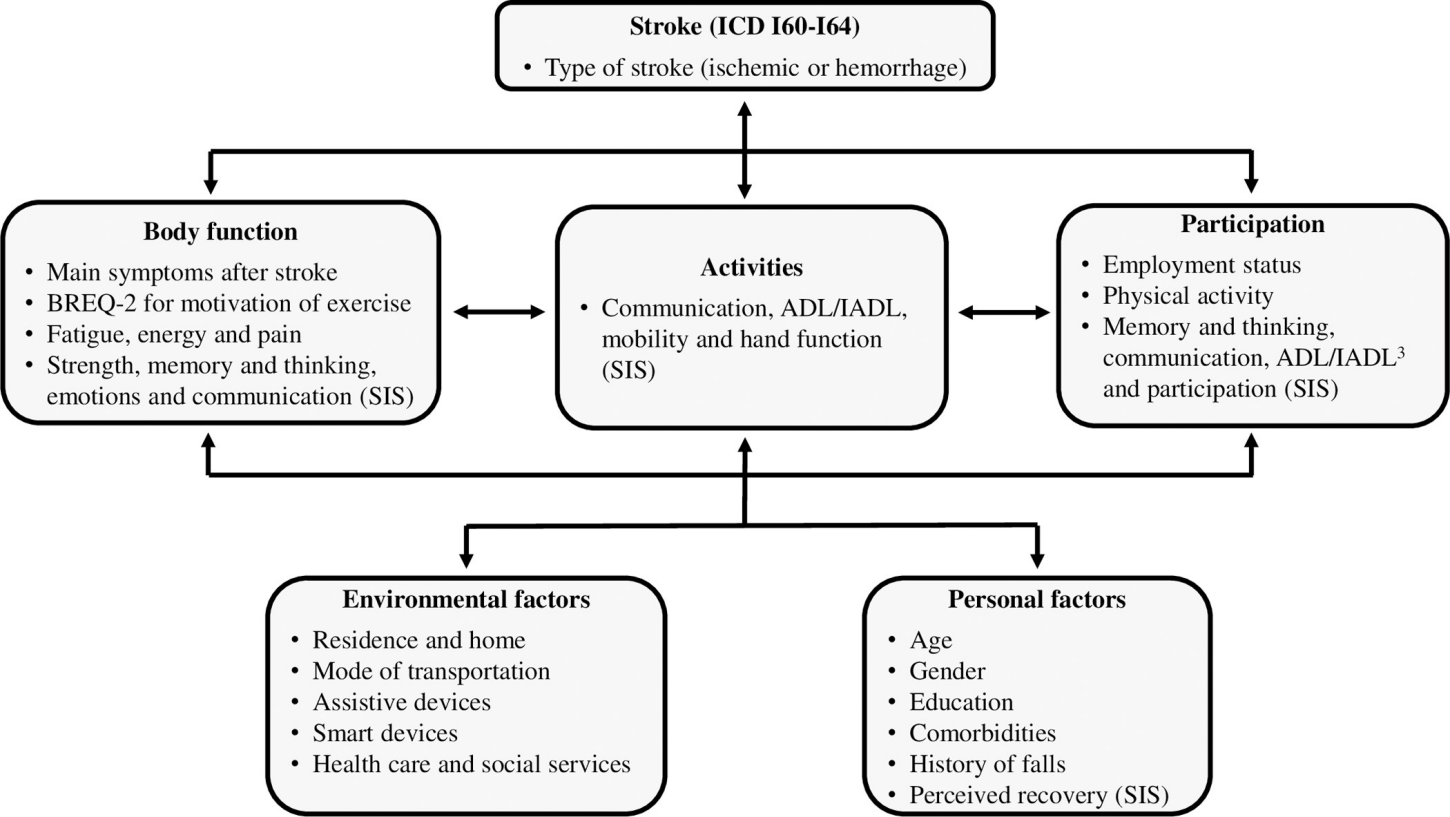
Linking of the questions in the survey to the components of the ICF framework.

We used the ICF Linking Rules [29–31] to link all survey items to the ICF and they covered all the ICF components except for *body structure* (Fig 1).

The use of ICF and standardized questionnaires allows our study to be compared effectively with international studies. The survey included 28 questions and two standardized instruments: the Stroke Impact Scale (SIS) [32] and the Behavioural Regulation Exercise Questionnaire 2 (BREQ-2) [33]. The SIS is an ICF-based stroke-specific health status measure which assesses perceived recovery along with eight domains of functioning: strength, memory and thinking, emotions, communication, activities of daily living (ADL)/instrumental activities of daily living (IADL), mobility, hand function, participation and perceived recovery [32]. Each SIS-domain includes a different number of questions (range 4–10). A total score for each domain can be calculated if participant responds to at least half of the questions, otherwise it is assigned as missing [34]. The total score for each SIS-domain range from 0 to 100 where zero is *an inability to complete the items* and 100 means *no difficulties experienced at all*. For perceived recovery zero equals *no recovery* and 100 *full recovery*. A composite physical domain can be created by summing the score from the domains for strength, hand function, mobility and ADL/IADL [34]. The SIS has shown good psychometric properties including validity [35,36], inter-rater/intra-rater reliability [37], test-retest reliability [34,38], and internal consistency [34]. It has also been tested for use as a mailed questionnaire showing high internal consistency [39]. The SIS has recently been translated into Icelandic using a translation/back-translation method [40]. The BREQ-2 assesses the motivation for exercise and includes 19 statements about engagement in exercise, scoring on a five-point Likert scale (0 = not true for me, 4 = very true for me). The BREQ-2 has five subscales: 1) amotivation, 2) external regulation, 3) introjected regulation, 4) identified regulation and 5) intrinsic regulation [33]. In line with the self-determined theory, *identified* and *intrinsic regulation* address self-determination (mean score range 0–8) while *amotivation*, *external regulation* and *introjected regulation* address non-self-determination (mean score range 0–12) [41]. Higher scoring of self-determination is positively linked with adaptive health behaviour, but higher scoring of non-self-determination indicates the opposite [42]. The psychometric properties of the BREQ-2 have been investigated in samples of healthy people [33,43] as well as in different patient groups [44,45]. To date, no data is available on psychometric properties when used for stroke survivors but the content and format supports its relevance within that group. Apart from the standardized instruments, a few of the questions were from existing instruments: a question on history of falls from the Prevention of Falls and Injury Trial [46], questions on fatigue and energy from the Fatigue Assessment Scale [47] and Fatigue Severity Scale [48] and a question on pain from EuroQol-5D [49].

The survey was self-reported but participants notified us by marking in an appropriate box if they received assistance. This assistance was allowed to optimize the participation rate and accuracy of responses among individuals with some writing, vision and/or minor communicative problems. The survey was pilot-tested on four community-dwelling stroke survivors (47–78 years old) who answered the final draft of the survey and gave feedback concerning clearer wording and options for answers.

### Procedure

The survey, along with an information letter and a stamped envelope for return, was sent to the eligible participants. As described in the information letter, participation was interpreted as giving informed consent. If eligible stroke survivors had not responded within three weeks, a researcher (SAO) followed up with a phone call. In the phone call the person was encouraged to take part and was offered assistance. Participants who refused to take part were politely asked to share the reason with the researcher.

The study was conducted according to the ethical principles of the Declaration of Helsinki and approvals were obtained from the Icelandic Data Protection Authorities and the Icelandic National Bioethics Committee (VSNb2017110024/03.01).

### Statistical methods

The R-statistical software was used for data analysis and the level of significance was set at *P*<0.05. No corrections were made for multiple statistical tests. In the BREQ-2, 42.1% of the data was missing. The missing data was influenced by age (t = 2.023) where younger stroke survivors were more likely to answer than the older ones. Therefore, imputation was used, using predictive mean matching [50]. This imputation method is less vulnerable to misspecification than other methods since there is no need to define an explicit model for the distribution of the missing values. The imputation process was completed with the statistical package ´mice´ in *R*, *statistical software* with random seed = 500. Age in years was used to create an ordinal variable with three categories: 75 years and older (≥75), 65–74 years and younger than 65 years (<65). Descriptive analysis included mean and 95% confidence interval for the continuous data, and frequencies and proportions based on valid answers for the categorical variables. Welch Two Sample t-test was used for comparing age of participants and non-participants and Chi-Square tests for genders and residence. For subgroup analysis by age, analysis of variance (ANOVA) was used for continuous data and Fisher´s exact tests for categorical variables. A post hoc text, TukeyHSD, was used for comparing possible age-group pairings.

## Results

### Participants

**Fig 2:**
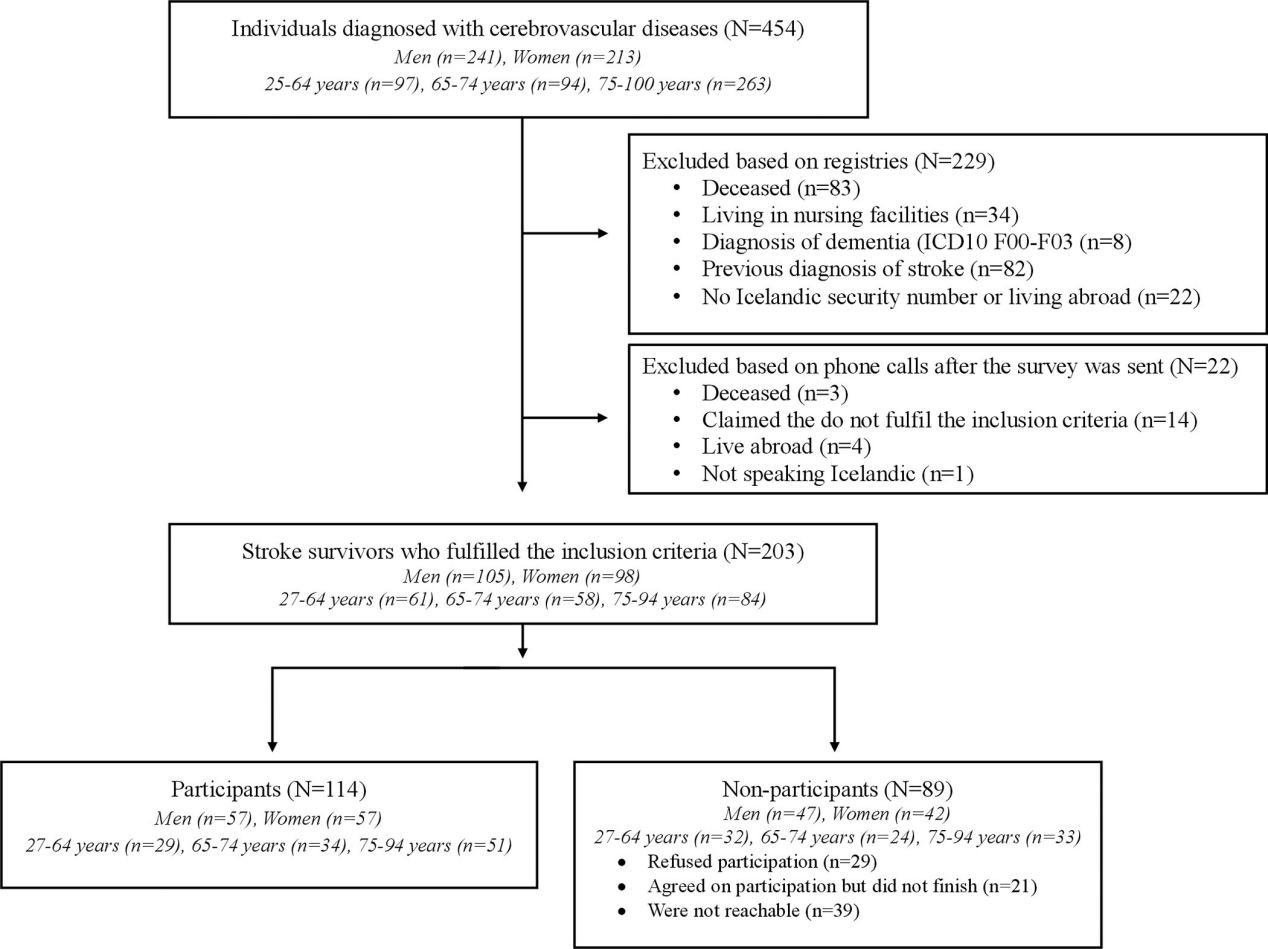
Flowchart of inclusion of participants.

A total of 454 individuals (men 53.1%) were admitted and diagnosed with a stroke (ICD10 I60-I64) within the pre-defined 12-month period (see flowchart in Fig 2). Eighty-six individuals had died (18.9%) but most stroke survivors were excluded due to a previous diagnosis of stroke (n = 82, 18.1%). Eligible participants were 203 (men 51.7%) and 114 participated (men 50%), resulting in a 56.2% response rate.

The participants were slightly older than the non-participants (mean age 71.6 ±12.9 years versus 68.1±13.5 years; P = 0.050, t = 1.96), and represented comparable residential areas (P = 0.834, χ2 = 0.04) and a comparable proportion of men and women (P = 0.798, χ2 = 0.06). Of the 78 individuals who received a phone call to facilitate their participation, 31 responded to the survey and 30 gave the following reasons for not participating: good/full recovery (n = 11), not interested (n = 7), difficult to remember the past (n = 6) and dependent on others (n = 6). Forty-one (36.0%) individuals received assistance with completing the survey, with more participants being ≥75 years old (*P* = 0.007) than younger than 65 years old.

The majority of participants (n = 74, 66.1%) reported having had an ischemic stroke, with no statistical difference between the age-groups (*P* = 0.735). Eighteen (15.8%) had had a haemorrhagic stroke, with no statistical difference between the age-groups (*P* = 0.052). Twenty participants (17.9%) stated they were unaware of the type of stroke they had had, with more participants older than 75 years old than younger than 65 years old (*P* = 0.003).

### Mapping of the ICF components

Personal factors. The mean age of the participants was 71.6 years (95% CI 69.2–74) with the median being 73 years (IQR 16.75; range 27–94 years). Fifty-one participants (44.7%) were ≥75 years old (men 45.1%), 34 (29.8%) were 65–74 years old (men 50%) and 29 (25.4%) were younger than 65 years old (men 58.6%), with no statistical difference between the genders in any of the age-groups (*P* = 0.519). The mapping of other personal factors is presented in Table 1.

**Table 1:** Personal factors.

	%^a^ (n) or Mean [95% CI]				
	All (N = 114)	75–94 years (n = 51)	65–74 years (n = 34)	27–64 years (n = 29)	p-value^b^
**Demography**					
Men	50.0% (57)	45.1% (23)	50.0% (17)	58.6% (17)	0.519
No postsecondary education	58.8% (67)	60.8% (31)	73.5% (25)	37.9% (11)	0.016^d^
**Main symptoms after the stroke** ^c^					
Balance impairments	61.4% (70)	60.8% (31)	76.5% (26)	44.8% (13)	0.038^e^
Aphasia	36.0% (41)	33.3% (17)	47.1% (16)	27.6% (8)	0.248
Memory impairments	32.5% (37)	35.3% (18)	32.4% (11)	27.6% (8)	0.789
Paresis/paralysis right UE	27.2% (31)	31.4% (16)	26.5% (9)	20.7% (5)	0.638
Paresis/paralysis left LE	26.3% (30)	27.5% (14)	35.3% (12)	13.8% (4)	0.134
Paresis/paralysis left UE	25.4% (29)	21.6% (11)	32.4% (11)	24.1% (7)	0.555
Apraxia	21.1% (24)	23.5% (12)	26.5% (9)	10.3% (3)	0.237
Paresis/paralysis right LE	18.4% (21)	13.7% (7)	20.6% (7)	24.1% (7)	0.479
Problems with swallowing	15.8% (18)	17.6% (9)	20.6% (7)	6.9% (2)	0.277
Neglect	12.3% (14)	11.8% (6)	17.6% (6)	6.9% (2)	0.469
Visual disturbances	5.3% (6)	2.0% (1)	8.8% (3)	6.9% (2)	0.322
Face numbness/paralysis	4.4% (5)	3.9% (2)	2.9% (1)	6.9% (2)	0.719
Headache	4.4% (5)	0% (0)	8.8% (3)	6.9% (2)	0.088
**Falls in the last 12 months**					
Experienced one or more fall	29.8% (34)	35.3% (18)	35.3% (12)	13.8% (4)	0.013^f^
Had fractures from falls	7.0% (8)	7.8% (4)	5.9% (2)	6.9% (2)	0.383
**Comorbidities**					
Number of comorbidities	1.5 [1.3–1.8]	2.0 [1.7–2.4]	1.4 [1.0–1.8]	0.8 [0.5–1.2]	< 0.001^g^
Cardiovascular diseases	50.9% (58)	62.7% (32)	38.2% (13)	44.8% (13)	0.067
Osteo-/Rheumatoid Arthritis	24.6% (28)	29.4% (15)	32.4% (11)	6.9% (2)	0.024^h^
Impaired urinary function	21.1% (24)	37.3% (19)	14.7% (5)	0% (0)	< 0.001^i^
Anxiety/depression	15.8% (18)	31.4% (16)	2.9% (1)	3.4% (1)	< 0.001^j^
Diabetes	12.3% (14)	11.8% (6)	20.6% (7)	3.4% (1)	0.140
Cancer	11.4% (13)	11.8% (6)	14.7% (5)	6.9% (2)	0.647
Osteoporosis	7.9% (9)	11.8% (6)	2.9% (1)	6.9% (2)	0.366
Myalgia	4.4% (5)	3.9% (2)	0% (0)	10.3% (3)	0.120
COPD	3.5% (4)	2.0% (1)	8.8% (3)	0% (0)	0.200

Abbreviations: UE = Upper extremity, LE = Lower extremity, No = Number, COPD = Chronic obstructive pulmonary disease.

^a^Proportions are based on valid data for each variable.

^b^Fisher´s Exact Test the categorical variables and Linear Model ANOVA for the continuous variable of number of comorbidities.

^c^The main symptoms after stroke were linked to personal factors as a lived experience, since the results reflected the current situation of participants, 1–2 years after stroke.

^d^Difference between 65–74 years old and <65 years old (p = 0.0152).

^e^Difference between 65–74 years old and <65 years old (p = 0.0316).

^f^Difference between ≥75 years old and <65 years old (p = 0.029).

^g^Difference between ≥75 years old and 65–74 years old (p = 0.029), ≥75 years old and <65 years old (p<0.001), and 65–74 years old and <65 years old (p = 0.035).

^h^Difference between ≥75 years old and <65 years old (p<0.001), and 65–74 years old and <65 years old (p = 0.013).

^i^Difference between ≥75 years old and 65–74 years old (p<0.001), ≥75 years old and <65 years old (p<0.001), and 65–74 years old and <65 years old (p = 0.032).

^j^Difference between ≥75 years old and 65–74 years old (p = 0.028), and ≥75 years old and <65 years old (p = 0.0431).

A difference was found among all three age-groups in the number of comorbidities, where the oldest individuals (≥75 years) had the most comorbidities and the youngest (<65 years) reported having the fewest. Cardiovascular disease was the most common diagnosis in all age-groups with no statistical difference between the age-groups (*P* = 0.067). The oldest individuals had more anxiety and depression than those in both younger age-groups.

#### Environmental factors

The mapping of environmental factors is presented in Table 2. The oldest individuals (≥75 years) had more walking devices (<65 years old *P* = 0.007, 65–74 years old *P* = 0.020) and more security buzzers (<65 years old *P* = 0.001, 65–74 years old *P*<0.001) than those in the younger age-groups. The majority of participants had access to smart devices, with computers being the most common. The oldest individuals had less access to computers (<65 years old *P* = 0.004, 65–74 years old *P* = 0.002) and smart phones (<65 years old *P*<0.001, 65–74 years old *P*<0.001) than those in the younger age-groups.

**Table 2:** Survey items linked to the ICF component of environmental factors.

	%^a^ (n)				
Environmental factors	All (N = 114)	75–94 years (n = 51)	65–74 years (n = 34)	27–65 years (n = 29)	p-value^b^
**Residence, housing and pension**					
Live in capital area (e215)	66.7% (76)	80.4% (41)	47.1% (16)	65.5% (19)	0.007^e^
Live alone (e398)	28.1% (32)	41.2% (21)	20.6% (7)	13.8% (4)	0.017^f^
Had to change housing after stroke (e155)	1.8% (2)	2.0% (1)	2.9% (1)	0% (0)	1.000
Good access in home (e155)	93.9% (107)	90.2% (46)	100% (34)	93.1% (27)	0.208
State pension^c^ (e570)	67.5% (77)	98% (50)	76.5% (26)	3.4% (1)	< 0.001^g^
Invalidity pension^d^ (e570)	8.8% (10)	0% (0)	0% (0)	34.5% (10)	< 0.001^h^
**Access to assistive and smart devices**					
Walking devices (e120)	28.9% (33)	47.1% (24)	17.6% (6)	10.3% (3)	< 0.001^i^
Wheelchairs (e120)	4.4% (5)	2.0% (1)	5.9% (2)	6.9% (2)	0.519
Buzzer (e115)	28.9% (33)	54.9% (28)	8.8% (3)	6.9% (2)	< 0.001^j^
Laptop or computer (e130)	68.4% (78)	47.1% (24)	85.3% (29)	86.2% (25)	< 0.001^k^
Smartphone (e130)	60.5% (69)	33.3% (17)	82.4% (28)	82.8% (24)	< 0.001^l^
Tablet (e130)	43.9% (50)	27.5% (4)	47.1% (16)	69.0% (20)	0.001^m^
**Health care and social services**					
Inpatient rehabilitation after stroke (e580)	84.2% (96)	82.4% (42)	85.3% (29)	86.2% (25)	1.000
Services during last month (e5)	45.6% (52)	62.7% (32)	41.2% (14)	20.7% (6)	0.001^f^
Fulfilled needs (n = 50) (e580)	66.0% (33)	63.3% (19)	69.2% (9)	71.4% (5)	1.000
Physical therapy (e580)	34.2% (39)	43.1% (22)	35.3% (12)	17.2% (5)	0.054
Occupational therapy (e580)	0.9% (1)	0% (0)	2.9% (1)	0% (0)	0.553
Speech therapy (e580)	0.9% (1)	2.0% (1)	0% (0)	0% (0)	1.000
Home nursing (e580)	7.0% (8)	9.8% (5)	5.9% (2)	3.4% (1)	0.648
Ambulant nursing(e580)	0.9% (1)	0% ()	2.9% (1)	0% (0)	0.533
Social domestic (e575)	14.0% (16)	25.5% (13)	2.9% (1)	6.9% (2)	0.006^n^
Adult day care (e580)	4.4% (5)	9.8% (5)	0% (0)	0% (0)	0.056^i^
Transportation services(e575)	6.1% (7)	11.8% (6)	0% (0)	3.4% (1)	0.071

^a^Proportions are based on valid data for each variable.

^b^Fisher´s Exact Test for categorical variables.

^c^State pension can be received at the age of 65.

^d^Personal Independence Payment can be received at the age of 18–66.

^e^Difference between ≥75 years old and 65–74 years old (p = 0.0054).

^f^Difference between ≥75 years old and <65 years old (p = 0.0407).

^g^Difference between ≥75 years old and 65–74 years old (p = 0.0321), ≥75 years old and <65 years old (p<0.001), and 65–74 years old and <65 years old (p = 0.0001).

^h^Difference between ≥75 years old and <65 years old (p<0.001), and 65–74 years old and <65 years old (p<0.001).

^i^Difference between ≥75 years old and 65–74 years old (p = 0.028), and ≥75 years old and <65 years old (p = 0.0431).

^j^Difference between ≥75 years old and 65–74 years old (p = 0.0199), and ≥75 years old and <65 years old (p = 0.0066).

^k^Difference between ≥75 years old and 65–74 years old (p = 0.0023), and ≥75 years old and <65 years old (p = 0.0038).

^l^Difference between ≥75 years old and 65–74 years old (p = 0.0001), and ≥75 years old and <65 years old (p = 0.0002).

^m^Difference between ≥75 years old and <65 years old (p = 0.0015).

^n^Difference between ≥75 years old and 65–74 years old (p<0.001), and ≥75 years old and <65 years old (p = 0.04).

#### Body function

Motivation for exercise, which was assessed with BREQ-2, showed more self-determination than non-self-determination in all age-groups. No statistical difference was found in self-motivation between the age-groups but the oldest age-group reported more non-self-determination than the youngest group (P = 0.034). No statistical differences were in found in other categories of body function between the age-groups (Table 3).

**Table 3:** Survey items linked to the ICF component of body function.

	Mean [95% CI] or %^a^ (n)				
Body function	All (N = 114)	75–94 years (n = 51)	65–74 years (n = 34)	27–64 years (n = 29)	p-value^b^
**BREQ-2 for motivation of exercise (**b130)					
Self-determination	4.8 [4.3–5.2]	4.9 [4.3–5.6]	4.8 [4.0–5.6]	4.6 [3.5–5.6]	0.786
Non-self-determination	3.0 [2.6–3.4]	3.3 (2.7–3.9)	3.3 [2.6–4.0]	2.1 [1.3–2.8]	0.028^c^
**I get tired very quickly** (b455)					0.189
Never or seldom	23.6% (25)	16.3% (8)	25.8% (8)	34.6% (9)	
Sometimes, most often or always	76.4% (81)	83.7% (41)	74.2% (23)	65.4% (17)	
**Fatigue is among my most disabling symptoms** (b455)					0.262
Never or seldom	43.0% (40)	34.1% (14)	46.4% (13)	54.2% (13)	
Sometimes, most often or always	57.0% (53)	65.9% (27)	53.6% (15)	45.8% (11)	
**I have enough energy for everyday life** (b130)					0.787
Never or seldom	15.4% (16)	18.2% (8)	12.5% (4)	14.3% (4)	
Sometimes, most often or always	84.6% (88)	81.8% (36)	87.5% (28)	85.7% (24)	
**Statements on pain today** (b280)					0.808
No or slight pain	75.5% (80)	71.7% (33)	78.8% (26)	77.8% (21)	
Moderate, severe or extreme pain	24.5% (26)	28.3% (13)	21.2% (7)	22.2% (6)	

^a^Proportions are based on valid data for each variable.

^b^Fisher´s Exact Test the categorical variables and Linear Model ANOVA for BREQ-2.

^c^Difference between ≥75 years old and <65 years old (p = 0.033).

#### Activities and participation

The mapping of activities and participation is presented in Table 4. The majority of participants (70.2%) drove a car with fewer individuals in the oldest group than in both younger groups (<65 years old *P* = 0.023, 65–74 years old *P* = 0.012). They also depended more on others for transportation than those younger than 65 years old (*P* = 0.031). Compared to both younger groups, the oldest individuals used computers less (<65 years old *P*<0.001, 65–74 years old *P*<0.001) and smart phones (<65 years old *P*<0.001, 65–74 years old *P* = 0.001).

**Table 4:** Survey items linked to the ICF component of activities and participation.

		%^a^ (n)			
Activities and participation	All (N = 114)	75–94 years (n = 51)	65–74 years (n = 34)	27–64 years (n = 29)	p-value^b^
**Employment status**					
Working full-time (d850)	14.9% (17)	2.0% (1)	8.8% (3)	44.8% (13)	< 0.001^d^
Working part-time (d850)	7.9% (9)	0% (0)	14.7% (5)	13.8% (4)	0.005^e^
Volunteering (d855)	4.4% (5)	3.9% (2)	5.9% (2)	3.4% (1)	1.000
**Transportation**					
Drive a car (d475)	70.2% (80)	56.9% (29)	79.4% (27)	82.8% (24)	0.023^f^
Depend on others^c^ (d470)	23.7% (27)	35.3% (18)	20.6% (7)	6.9% (2)	0.019^g^
Use public transport (d470)	7.9% (9)	11.8% (6)	2.9% (1)	6.9% (2)	0.366
**Regular use of smart devices**					
Laptop or computer (d369)	54.4% (62)	27.5% (14)	73.5% (25)	79.3% (23)	< 0.001^h^
Smartphone (d369)	51.8% (59)	27.5% (14)	67.6% (23)	75.9% (22)	< 0.001^i^
Tablet (d369)	33.3% (38)	17.6% (9)	41.2% (14)	51.7% (15)	0.003^j^
**Physical activity or exercise**					
At least three times a week (d570)	64.0% (73)	58.8% (30)	70.6% (24)	65.5% (19)	0.559
At least five times a week (d570)	47.4% (54)	45.1% (23)	50.0% (17)	48.3% (14)	0.888

^a^Proportions are based on valid data for each variable.

^b^Fisher´s Exact Test for categorical variables.

^c^Includes depending on individuals as well as use of transportation services for disabled.

^d^Difference between ≥75 years old and <65 years old (p = 0.002), and 65–74 years old and <65 years old (p = 0.008).

^e^Difference between ≥75 years old and <65 years old (p = 0.027).

^f^Difference between ≥75 years old and 65–74 years old (p = 0.012), and ≥75 years old and <65 years old (p = 0.023).

^g^Difference between ≥75 years old and <65 years old (p = 0.0416).

^h^Difference between ≥75 years old and 65–74 years old (p = 0.0002), and ≥75 years old and <65 years old (p = 0.0001).

^i^Difference between ≥75 years old and 65–74 years old (p = 0.0012), and ≥75 years old and <65 years old (p = 0.0002).

^j^Difference between ≥75 years old and <65 years old (p = 0.0059).

### Stroke impact scale

The results from the SIS are presented in Table 5. The highest score was in the communication domain and the lowest score was in the emotion domain. Differences were found between the age-groups in three domains: ADL/IADL (*P* = 0.002), mobility (*P*<0.001) and participation (*P* = 0.020) as well as in the composite physical domain (*P* = 0.040). The oldest individuals (≥75 years) deviated from the two younger age-groups in ADL/IADL (<65 years old *P* = 0.001, 65–74 years old *P* = 0.037) and mobility (<65 years old *P*<0.001), 65–74 years old *P* = 0.016) and from the youngest group (<65 years) in participation (*P* = 0.005), and composite physical domain (*P* = 0.015).

**Table 5:** Results from stroke impact scale.

		Mean [95% CI]			
Domains of Stroke Impact Scale (ICF code)	All (N = 114)	75–94 years (n = 51)	65–74 years (n = 34)	27–65 years (n = 29)	p-value^a^
**Strength** (b730)	72.8 [68.2–77.4]	70.6 [63.7–77.5]	69.0 [60.7–77.2]	81.5 [72.1–90.9]	0.096
**Memory and thinking** (b114, b140, b144, b160, d230)	77.9 [73.3–82.6]	74.9 [66.8–83.0]	78.7 [72.5–84.9]	82.7 [73.5–91.8]	0.403
**Emotions** (b152)	65.0 [62.1–67.8]	63.3 [59.1–67.5]	65.6 [60.0–71.2]	67.2 [61.2–73.2]	0.530
**Communication** (b167, d350, d360)	83.3 [79.0–87.5]	80.1 [72.9–87.3]	84.9 [77.9–91.9]	87.0 [78.9–95.1]	0.387
**ADL/IADL** (b525, b620, d510, d520, d530, d540, d550, d620, d640)	80.3 [76.5–84.2]	75.0 [69.1–81.0]	79.7 [73.2–86.2]	91.5 [85.3–97.8]	0.002^c^
**Mobility** (d410, d415, d450, d455)	77.5 [73.2–81.8]	70.3 [63.3–77.3]	78.9 [73.0–84.7]	90.1 [82.8–97.3]	<0.001^d^
**Hand function** (d430, d440, d445)	77.7 [72.1–83.3]	73.9 [65.0–82.8]	76.2 [66.9–85.4]	87.0 [75.4–98.6]	0.167
**Participation** (d750, d760, d850, d855, d920, d930)	73.2 [68.0–78.5]	67.5 [58.6–76.3]	71.8 [63.1–80.5]	85.5 [77.6–93.3]	0.020^e^
**Perceived recovery** (personal factor)	74.8 [70.7–78.8]	71.8 [64.7–78.8]	73.1 [66.6–79.5]	81.4 [73.9–88.9]	0.143
**Composite physical domain**^b^	75.2 [71.2–79.1]	71.0 [65.2–76.8]	74.7 [68.8–80.7]	83.5 [74.1–92.9]	0.040^f^

^a^Linear Model ANOVA.

^b^Composite physical domain includes the domains of strength, ADLs/IADLs, mobility, and hand function.

^c^Difference between ≥75 years old and 65–74 years old (p = 0.037), and ≥75 years old and <65 years old (p = 0.001).

^d^Difference between ≥75 years old and 65–74 years old (p = 0.016), and ≥75 years old and <65 years old (p<0.001).

^e^Difference between ≥75 years old and <65 years old (p = 0.005).

^f^Difference between ≥75 years old and <65 years old (p = 0.015).

## Discussion

Our results highlight functioning and contextual factors among community-dwelling stroke survivors one to two years after their first stroke based on the ICF. Some interesting differences between the three specific age-groups of stroke survivors include the number of comorbidities, access to and use of smart devices and scoring in the SIS-domains of ADLs/IADLs and participation. At the same time, these age-groups were noticeably similar in their adherence to physical activity and in the SIS-domain of memory and thinking. Although differences were most notable between the oldest (≥75 years old) and the youngest group, (<65 years old), there were some important differences between the two older groups indicating more impairments and showing that more support is needed among the oldest individuals (≥75 years old). At the same time there were some noteworthy similarities between the two younger groups which indicate high functioning of these two age-groups. In line with studies within gerontology, these results support the need for exploring functioning among stroke survivors who are older than 65 years of age in different age-groups.

In our study, the ICF was used to organize the complex pattern of functioning and disability one to two years after first stroke and to map contextual factors of community-dwelling stroke survivors. Moreover, we used the linking rules [29–31] to code all the variables from our survey and link them to the appropriate ICF categories, and thereby we transformed our results to the international language of the ICF. Other national surveys have been conducted with different survey items and different time points post-stroke [12,16,17,20,21] and linking their variables to the ICF would improve the potential for international comparisons.

In studies focusing on potential age differences among stroke survivors, the age-groups and analysis fluctuate markedly [4,5,18–21,51], which makes comparisons of results difficult, but in general, worse functioning is associated with higher age. In a study on stroke survivors, 10 years after stroke, increased age was correlated with less functioning and more disability, when four specific age-groups were compared (<65 years, 65–74 years, 75–84 years and ≥75 years) but differences between the groups were not analysed [20]. In a study on participation of stroke survivors younger and older than 70 years old, the older group had significantly more restrictions in participation due to impaired mobility [18]. A study on stroke survivors showed that age was significantly associated with care dependency, and those who were older than 75 years old had more risk of care dependency than stroke survivors 75 years and younger [19].

Use of technical solutions through smart devices is increasing in rehabilitation among community-dwelling stroke survivors [52–55] and facilitates participation after stroke [56]. Studies have shown that they are interested in using smart devices for exercise and physical activity, especially those who have an experience of using smart devices [57,58]. Our results show that the majority of participants had access to a smart device, which provides good opportunities to approach community-dwelling stroke survivors with different rehabilitation interventions in their own urban and rural homes. This good access is also highly relevant during COVID times where physical distancing is the main issue [7]. As could be expected from a population furthest from being “digital natives”, the oldest participants (≥75 years) reported the least access and use of smart devices. Yet almost half of this age-group had access to smart devices, which may indicate a potential for future use in community-based rehabilitation, given appropriate support.

The response rate of our survey was 56.2%, which is considered acceptable to good for a mailed population-based survey where the potential participants are identified from registries that cover all citizens diagnosed with stroke [59]. In previous surveys on stroke survivors, participants have been recruited from stroke clinics and support groups and showed a wide response rate, in the range 17–78% [16,17,21]. Surveys that recruit individuals from support groups and volunteers tend to have higher response rates than surveys where participants are identified through registries like in our study [21]. Our total population sample and acceptable participation rate strengthen the generalizability of the results for community-dwelling first-time stroke survivors, without the diagnosis of dementia, in Iceland and might give indications for comparable populations in other nations.

Successful community-based rehabilitation and integration must be tailored to the individuals needs and directed toward maximizing stroke survivors´ functioning, which, according to the ICF, is based on a dynamic interaction between stroke survivors´ health condition and contextual factors. When exploring the influence of age and ageing, stroke survivors are commonly divided into two groups only; younger than 65 years old and 65 years and older [21,22]. Our results, however, support that older stroke survivors (65 years and older) are too heterogeneous to be treated as one group. At the same time, additional heterogeneity likely exists in the groups of those 65 years and younger and 75 years and older. Moreover, our findings reveal the need to direct the focus of community-based rehabilitation and research towards the heterogeneous group of older stroke survivors in order to discover how to best meet their requirements and promote community integration.

Our study has a few limitations and strengths that should be mentioned. As our study was based on a total population sample, power analysis was not conducted. The target sample was based on thorough hospital registries of the two main hospitals in Iceland, where nearly all individuals who experience a stroke in Iceland are admitted. The small sample size, mainly due to the small population of the Icelandic nation, limited the power of the study, resulting in possible underestimation (Type II error) of differences between the age-groups. Yet the study was powered enough to detect some interesting age-group differences and provide ICF mapping of the functioning of community-dwelling first-stroke survivors in this small nation. These findings may be generalizable to other contexts where rehabilitation pathways and access to smart devices are comparable. Future research with larger samples may extend our analysis by applying multivariable approach, adjusting for possible confounding factors, and potentially detect further differences between age-groups of interest. Importantly, larger samples would also give the possibility to explore and understand potential heterogeneity within the age-groups. In addition, further research including data from the first year following stroke, would be needed to identify if the trends and differences between age-groups persist at earlier time points following stroke.

Although the participation rate was acceptable, it is important to consider the possibility of non-response bias with approximately 43% of the eligible sample not participating. The participants were slightly older than the non-participants, but information gathered from the follow-up calls on 30 of the 89 non-participants, did not indicate any obvious systematic differences in recovery or functioning (at the level of body function, activities or participation) between participants and non-participants. When analyzing the data, we were not able to account for stroke severity since such information was not collected in the study. At the same time, the participants were most likely to have had mild or moderate strokes as all of them were discharged to their home and lived in the community for 1–2 years after the stroke. About one third of the participants received some help with completing the survey which may have prompted responses that were desirable to the proxy as opposed to an accurate response from the participant. However, this might have given us answers from older participants and/or participants with writing, vision, or communicative problems.

## Supporting information

S1 File (PDF)

S2 File (PDF)

S1 Data (XLSX)
