# Supplementary material for: Age differences in functioning and contextual factors in community-dwelling stroke survivors: A national cross-sectional survey
Source: PLoS One. 2022 Aug 25;17(8):e0273644. doi: 10.1371/journal.pone.0273644 (PMC9409552; doi:10.1371/journal.pone.0273644)
Supplement: S1 File — (PDF) [file pone.0273644.s001.pdf]

Question	Data format
Yes, I did receive assistance with answering the quiestions.	1=yes
How old are you?	xx
	1=male<br>2=female
What is the postal code of your residency?	xxx
How many persons live in your home, including yourself?	1=I live alone<br>2=Two<br>3=Three or more
What is the highest level of education you have finished?	1= compulsory education<br>2= Final exam at high school, secondary school certificate<br>3= Vocational education<br>4=University degree<br>5=other education
What was the reason for your stroke?	1= blood clot in brain<br>2=bleeding in brain<br>3=I don´t know
**What were the main symtoms after the stroke?**	
Other symptoms, what?	
paresis/paralysis of right arm (1)	1=yes
paresis/paralysis of right hand (2)	1=yes
paresis/paralysis of right leg (3)	1=yes
paresis/paralysis of right foot (4)	1=yes
paresis/paralysis of left arm (5)	1=yes
paresis/paralysis of left hand (6)	1=yes
paresis/paralysis of left leg (7)	1=yes
paresis/paralysis of left foot (8)	1=yes
balance impairment (9)	1=yes
apraxia (10)	1=yes
apraxia (11)	1=yes
Neglegt (12)	1=yes
problems with swallowing (13)	1=yes
loss of memory (14)	1=yes
other symptoms (15)	1=yes
**Comorbities?**	
Cardiovascular diseases	1=yes
diabetis	1=yes
Anxiety or depression	1=yes
COPD	1=yes
Osteoarthritis or RA	1=yes
osteoporosis	1=yes

Question	Data format
cancer	1=yes
Urinary incontinence	1=yes
**What was your employment status prior to the stroke?**	
I was working full-time	1=yes
I was working part-time	1=yes
I was a student	1=yes
I was retired (due to age)	1=yes
I was unemployed	1=yes
I was a volunteer	1=yes
What is your employment status as of now?	
I am working full-time	1=yes
I am working part-time	1=yes
I am a student	1=yes
I am a volunteer	1=yes
I am retired (due to age)	1=yes
I am unable to work	1=yes
I can not find a job that fits my function	1=yes
**Transport and devices**	
Did you have to change your place of living after the stroke because of unsuitable access?	1=no<br>2=yes
What was your primary mode of transport prior to your stroke?	
I drove a car	1=yes
I used public transport/taxi	1=yes
I used transport service for disabled	1=yes
I was dependent on others	1=yes
What is your primary mode of transport as of now?	
I drive a car	1=yes
I use public transport/taxi	1=yes
I use transport service for disabled	1=yes
I am dependent on others	1=yes
Do you use assistive devices?	1=no, I don´t use AD<br>2=yes
A cane or crutches	1=inside<br>2=outside
A walker	1=inside<br>2=outside
Regular wheelchair	1=inside<br>2=outside
Electric wheelchair	1=inside<br>2=outside
A scooter	1=inside<br>2=outside

Question	Data format
Other assistive devices for activities of daily living (i.e. sock/stocking aid, reacher aid or raised toilet seat)	1=inside<br>2=outside
Do you have a personal security buzzer?	1=no<br>2=yes
I have my own or have access to . . .	1-3 in the same column
Smart phone (1)	1=yes
Tablet (2)	1=yes
Laptop/computer (3)	1=yes
I use on regular basis . . .	1-3 in the same column
Smart phone (1)	1=yes
Tablet (2)	1=yes
Laptop/computer (3)	1=yes
**Services**	
Did you receive inpatient rehabilitation after the stroke?	1=no<br>2=yes, right after hospitalization<br>3=yes, after being discharged to my home
What service have you received during the last month and what has it included?	
physical therapy	1=yes
occupational therapy	1=yes
speech therapy	1=yes
ambulant nursing	1=yes
home nursing	1=yes
home service from the municipality	1=yes
day clinic /day care	1=yes
Does the service you have had in the last month fullfil your needs?	1=yes<br>2=no
**Are you physically active or do you exercise on regular basis?**	
no	1=no
yes, in a gym/sport center	1=yes
How often in a week?	xx
