# Supplementary material for: Age differences in functioning and contextual factors in community-dwelling stroke survivors: A national cross-sectional survey
Source: PLoS One. 2022 Aug 25;17(8):e0273644. doi: 10.1371/journal.pone.0273644 (PMC9409552; doi:10.1371/journal.pone.0273644)
Supplement: S2 File — (PDF) [file pone.0273644.s002.pdf]

HÁSKÓLI ÍSLANDS

LANDSPÍTALI HÁSKÓLASJÚKRAHÚS

SAk SJÚKRAHÚSIÐ Á AKUREYRI AKUREYRI HOSPITAL

Heilsa, færni og aðstæður

Einstaklingar í heimahúsum eftir eitt heilaslag

Háskóla Íslands, apríl 2018

Kæri þátttakandi

Takk fyrir að gefa þér tíma til að svara þessari könnun. Við viljum minna þig á að lesa ítarlega leiðbeiningar fyrir hverja spurningu, því svarmöguleikar eru mismunandi, og svara síðan spurningunum eftir bestu getu. Mundu að ekkert svar er réttara en annað. Við viljum fyrst og fremst fá að vita hvaða svar þér finnst lýsa best þér og þinni reynslu.

Við viljum jafnframt nefna aftur að algjör nafnleynd verður viðhöfð. Nafn þitt eða aðrar upplýsingar sem benda til þess hver þú ert, munu hvergi koma fram þegar niðurstöður þessarar rannsóknir verða birtar.

Við viljum biðja þig um að merkja við hér ef þú hefur fengið aðstoð við að svara spurningunum.

☐ Já, ég fékk aðstoð við að svara spurningunum.

Eitthvað sem þú vilt taka fram:

____

____

____

Bestu þakkir fyrir þátttökuna

Steinunn A. Ólafsdóttir sjúkraþjálfari í doktorsnámi við Háskóla Íslands (HÍ)
Ingibjörg Hjaltadóttir dósent við hjúkrunarfræðideild HÍ, ábyrgðarmaður rannsóknarinnar
Sólveig Ása Árnadóttir dósent við námsbraut í sjúkraþjálfun HÍ
Helga Jónsdóttir prófessor við hjúkrunarfræðideild HÍ
Þóra Berglind Hafsteinsdóttir prófessor við hjúkrunarfræðideildi HÍ

A. Lýðfræði- og félagslegar upplýsingar

A1. **Hvað ert þú gamall/gömul?** ___ **ára**

A2. **Kyn** ☐ Karl ☐ Kona

A3. **Í hvaða póstnúmeri býrð þú?** ___

A4. **Hversu margir búa á heimili þínu að þér meðtöldum/meðtalinni?**

- ☐ ég bý ein/einn
- ☐ tveir
- ☐ þrír eða fleiri

A5. **Hvert er hæsta stig menntunar sem þú hefur lokið?**

- ☐ skyldunám (t.d. barnaskólapróf, grunnskólapróf, gagnfræðapróf, landspróf)
- ☐ stúdentspróf eða annað próf á framhaldsskólastigi
- ☐ iðnnám eða tækniskólapróf
- ☐ háskólapróf (BS/BA, MS/MA, PhD)
- ☐ önnur menntun, hver? ______

B. Heilsufar, færni og líðan

B1. **Hvenær fékkst þú heilaslag?** *Ritaðu mánuðinn og árið.* ______

B2. **Hver var orsök heilaslagsins?**

- ☐ blóðtappi í heila
- ☐ blæðing í heila
- ☐ veit það ekki

B3. **Hver voru helstu einkennin eftir heilaslagið?** *Merktu við allt sem við á.*

- ☐ máttminnkun/lömun í hægri handlegg
- ☐ máttminnkun/lömun í hægri hendi
- ☐ máttminnkun/lömun í hægri fótlegg
- ☐ máttminnkun/lömun í hægri fæti
- ☐ máttminnkun/lömun í vinstri handlegg
- ☐ máttminnkun/lömun í vinstri hendi
- ☐ máttminnkun/lömun í vinstri fótlegg
- ☐ máttminnkun/lömun í vinstri fæti
- ☐ jafnvægisskerðing
- ☐ verkstol
- ☐ gaumstol
- ☐ málstol
- ☐ kyngingarörðugleikar
- ☐ minnisleysi
- ☐ önnur einkenni, hver? ______

B4. **Hefur þú greinst með aðra sjúkdóma eða kvilla?** *Merktu við allt sem við á.*

- ☐ hjarta- og æðasjúkdóma
- ☐ sykursýki
- ☐ kvíða eða þunglyndi
- ☐ langvinna lungnateppu
- ☐ slitgigt eða liðagigt
- ☐ beinþynningu
- ☐ krabbamein
- ☐ þvagleka
- ☐ annað, hvað ______________________________

B5. **Eftirfarandi staðhæfingar eru um þreytu og orku**. *Merktu við það sem þér finnst eiga best við þig við hverja staðhæfingu.*

	**Aldrei**	**Einstöku sinnum**	**Stundum**	**Oftast**	**Alltaf**
Ég þreytist mjög fljótt	☐	☐	☐	☐	☐
Ég hef næga orku til að komast í gegnum daginn	☐	☐	☐	☐	☐
Þreyta er mín mesta fötlun	☐	☐	☐	☐	☐

B6. **Eftirfarandi staðhæfingar eru um verk**i. *Merktu við það sem þér finnst eiga best við þig í dag.*

- ☐ Ég finn hvorki fyrir verkjum né óþægindum
- ☐ Ég finn fyrir vægum verkjum eða óþægindum
- ☐ Ég finn fyrir miðlungs miklum verkjum eða óþægindum
- ☐ Ég finn fyrir miklum verkjum eða óþægindum
- ☐ Ég finn fyrir óbærilegum verkjum eða óþægindum

B7. **Á síðustu 12 mánuðum, hefur þú dottið þannig að þú hafir lent á jörðinni eða gólfinu ?**

- ☐ nei
- ☐ já   Hversu oft hefur þú dottið á síðustu 12 mánuðum? _______

  Beinbrotnaðir þú eða hlaust alvarlegar tognanir við byltu sem hafði áhrif á daglega færni þína?

  - ☐ nei
  - ☐ já

C. Umhverfi og aðstæður

C1. **Hvernig var störfum þínum háttað áður en þú fékkst heilaslag**? *Merktu við allt sem við á.*

- ☐ Ég var í fullu starfi
- ☐ Ég var í hlutastarfi
- ☐ Ég var í námi
- ☐ Ég var ekki í vinnu sökum aldurs
- ☐ Ég var atvinnulaus
- ☐ Ég sinnti sjálfboðaliðastarfi
- ☐ Annað, hvað ____________________

C2. **Hvernig er störfum þínum háttað í dag**? *Merktu við allt sem við á.*

- ☐ Ég er í fullu starfi
- ☐ Ég er í hlutastarfi
- ☐ Ég er í námi
- ☐ Ég er ekki í vinnu vegna aldurs
- ☐ Ég er ekki í vinnu því ég er ekki fær um það
- ☐ Ég er ekki í vinnu því ég fæ ekki vinnu við hæfi
- ☐ Annað, hvað ____________________

C3. **Þurftir þú að skipta um húsnæði eftir heilaslagið vegna aðgengis** ?

- ☐ nei
- ☐ já

C4. **Hvernig er aðgengi fyrir þig að heimili þínu í dag**? *Merktu við það sem lýsir best aðstæðum á heimili þínu.*

- ☐ Gott, þarf ekki að fara stiga
- ☐ Gott, þarf að fara stiga og kemst auðveldlega milli hæða
- ☐ Ekki gott, þarf að fara stiga og á erfitt með að fara á milli hæða

C5. **Hvernig var ferðamáti þinn áður en þú fékkst heilaslag**? *Merktu við allt sem við á.*

- ☐ Ég ók bíl
- ☐ Ég notaði strætó/almenningssamgöngur/leigubíl
- ☐ Ég notaði Ferðaþjónustu fatlaðra
- ☐ Ég var háður öðrum en Ferðaþjónustu fatlaðra með ferðir

C6. **Hvernig er ferðamáti þinn í dag** ? *Merktu við allt sem við á.*

- ☐ Ég ek bíl
- ☐ Ég nota strætó/almenningssamgöngur/leigubíl
- ☐ Ég nota Ferðaþjónustu fatlaðra
- ☐ Ég er háður öðrum en Ferðaþjónustu fatlaðra með ferðir

C7. **Notar þú hjálpartæki?** *Merktu við allt sem við á.*

☐ nei, ég nota engin hjálpartæki

☐ já

	Innanhúss	Utanhúss
Staf eða hækjur	☐	☐
Göngugrind	☐	☐
Handknúinn hjólastól	☐	☐
Rafmagnshjólastól	☐	☐
Rafskutlu	☐	☐
Önnur hjálpartæki við athafnir daglegs lífs (t.d. sokkaífæru, griptöng eða salernisupphækkun)	☐	☐

C8. **Ertu með öryggishnapp t.d. frá Securitas eða Öryggismiðstöðinni?**

☐ nei

☐ já

C9. **Hér er spurt um aðgengi þitt og notkun á snjalltækjum.** *Merktu við allt sem við á.*

	snjallsíma	spjaldtölvu	borðtölvu/ fartölvu
Ég á eða hef aðgengi að ...	☐	☐	☐
Ég nota reglulega ...	☐	☐	☐

Hvernig / til hvers notar þú þessi snjalltæki? ______________________________

____

____

	snjallsíma	spjaldtölvu	borðtölvu/ fartölvu
Ég tel að að þessi tæki geti nýst mér til þjálfunar heimavið.	☐	☐	☐

Hvernig telur þú að þessi snjalltæki gætu nýst þér til þjálfunar? ______________________

____

____

D. Þjónusta og hreyfing

D1. **Fórstu á stofnun til endurhæfingar eftir heilaslagið (t.d. Grensás, Landakot, Reykjalund eða Kristnes) ?**

- ☐ nei
- ☐ já, strax eftir sjúkrahúsdvöl
- ☐ já, en fór heim í millitíðinni

Hvað leið langur tími þar til þú fórst í endurhæfingu (í dögum, vikum eða mánuðum)? ____________

Hvert fórstu í endurhæfingu? ____

Hvað dvaldir þú lengi þar (í vikum eða mánuðum)? ______________________________

D2. **Hvaða þjónustu fékkstu <u>strax</u> eftir útskrift af sjúkrahúsi eða endurhæfingarstofnun?**
Merktu við allt sem við á.

- ☐ sjúkraþjálfun
- ☐ iðjuþjálfun
- ☐ talþjálfun
- ☐ göngudeildarþjónustu hjúkrunarfræðinga
- ☐ heimahjúkrun (t.d. aðstoð við lyf, aðstoð við klæðnað eða böðun)
- ☐ heimaþjónustu sveitarfélags (t.d. þrif eða heimsendan mat)
- ☐ dagþjónustu (t.d. hjá Sjálfsbjörg eða á öldrunarheimilum)
- ☐ annað, hvað? ____

D3. **Hvaða þjónustu hefur þú fengið <u>síðastliðinn mánuð</u> og í hverju hefur hún falist?** *Merktu við allt sem við á og svaraðu viðeigandi spurningum.*

☐ **sjúkraþjálfun** hversu oft í viku? ____________

í hverju felst þjálfunin? ____________

☐ **iðjuþjálfun** hversu oft í viku? ____________

í hverju felst þjálfunin? ____________

☐ **talþjálfun** hversu oft í viku? ____________

í hverju felst þjálfunin? ____________

☐ **göngudeildarþjónusta hjúkrunarfræðinga** hversu oft í viku? ____________

í hverju felst þjónustan? ____________

☐ **heimahjúkrun** hversu oft í viku? ____________

í hverju felst þjónustan? ____________

☐ **heimaþjónusta sveitarfélags** hversu oft í viku? ____________

í hverju felst þjónustan? ____________

☐ **dagþjónustu** hversu oft í viku? ____________
(*t.d hjá Sjálfsbjörgu eða á öldrunarstofnunum*)

í hverju felst þjónustan? ____________

D4. **Uppfyllir sú þjónusta sem þú hefur fengið síðastliðinn mánuð þarfir þínar?**

☐ já
☐ nei

Hvernig myndir þú vilja bæta/breyta þjónustunni? ________________________________

____

____

Annað sem þú vilt taka fram um þjónustu:________________________________

____

____

D5. **Hefur þú gengið í að minnsta kosti 10 mínútur samfellt einhvern daginn á síðustu sjö dögum?**

☐ nei
☐ já — Hversu marga daga af síðustu sjö dögum varstu á göngu í að minnsta kosti 10 mínútur samfellt? ____________________

Hvað gekkstu lengi í hvert sinn að jafnaði ? ____________________

D6. **Hvað situr þú lengi á hverjum degi að jafnaði?** *Miðaðu við síðustu sjö daga.*

____

D7. **Stundar þú reglulega líkamsrækt eða æfingar?** *Merktu við allt sem við á.*

		Hversu oft í viku?
☐	nei	
☐	já, á líkamsræktarstöð	________
☐	já, í skipulagðri hópþjálfun t.d. á sjúkraþjálfunarstöð, HL-stöð eða félagsmiðstöð	________
☐	já, ég fer í sundleikfimi og/eða syndi	________
☐	já, ég geri æfingar heima	________
☐	já, annað, hvað?	________

D8. **Hvers vegna gerir þú æfingar eða ekki?** Okkur leikur hugur á að vita hvað liggur að baki ákvörðun einstaklinga um að gera æfingar eða ekki. Merktu við tölustaf (0-4) sem lýsir best hvernig eftirfarandi staðhæfingar eiga við þig

	**Á ekki við mig**		**Á stundum við mig**		**Á mjög vel við mig**
Ég geri æfingar af því að aðrir segja að ég eigi að gera þær	0	1	2	3	4
Ég fæ samviskubit þegar ég geri ekki æfingar	0	1	2	3	4
Ég met gagnsemi eða ávinning æfinga	0	1	2	3	4
Ég geri æfingar af því að það er gaman	0	1	2	3	4
Ég sé ekki ástæðu til að gera æfingar	0	1	2	3	4
Ég geri æfingar af því að vinir/fjölskylda segja að ég eigi að gera þær	0	1	2	3	4
Ég skammast mín þegar ég geri ekki æfingar	0	1	2	3	4
Það er mikilvægt fyrir mig að gera æfingar reglulega	0	1	2	3	4
Ég skil ekki af hverju ég ætti að gera æfingar	0	1	2	3	4
Ég nýt þess að gera æfingar	0	1	2	3	4
Ég geri æfingar af því að aðrir verða óánægðir með mig ef ég geri þær ekki	0	1	2	3	4
Ég sé ekki tilgang með æfingum	0	1	2	3	4
Mér finnst ég hafa brugðist ef ég geri ekki æfingar í nokkurn tíma	0	1	2	3	4
Ég tel mikilvægt að leggja mig fram við að gera æfingar reglulega	0	1	2	3	4
Mér finnst skemmtilegt að gera æfingar	0	1	2	3	4
Ég finn fyrir þrýstingi frá vinum/fjölskyldu að gera æfingar	0	1	2	3	4
Ég verð eirðarlaus ef ég geri ekki æfingar reglulega	0	1	2	3	4
Ég finn fyrir gleði og ánægju við að gera æfingar	0	1	2	3	4
Mér finnst það tímaeyðsla að gera æfingar	0	1	2	3	4

Mælistika á áhrif heilaslags

Tilgangur spurningalistans er að meta hvernig heilaslagið hefur haft áhrif á heilsu þína og líf. Okkur langar að fá að vita hvernig heilaslagið hefur haft áhrif á þig, út frá ÞÍNU SJÓNARHORNI. Eftirfarandi spurningar eru um skerðingu og fötlun sem heilaslagið hefur mögulega valdið, og einnig um hvernig heilaslagið hefur haft áhrif á lífsgæði þín. Að lokum, viljum við biðja þig að meta hversu mikinn bata þér finnst þú hafa fengið eftir heilaslagið.

Spurningar 1-8 fela í sér nokkra liði hver, þar sem boðið er upp á fimm svarmöguleika. Merktu við tölustaf (1-5) til að svara. Í spurningu 9 er kvarðinn 0-100 og þar merkir þú á kvarðann svar þitt.

© dr. Pamela Duncan og félagar, University of Kansas, Medical Center. Umsjón með íslenskri þýðingu 2017:
Sif Gylfadóttir, sjúkraþjálfari MSc, Reykjalundi, netfang: sifg@reykjalundur.is. Þýtt með leyfi höfundar.

1. **Eftirfarandi spurningar eru um líkamleg vandamál sem hafa mögulega komið upp vegna heilaslagsins.** ***Spurningarnar eiga við síðastliðna viku.***

**Hvernig myndir þú meta kraft þinn í ...**	**Mikill kraftur**	**Fremur mikill kraftur**	**Miðlungs kraftur**	**Lítill kraftur**	**Enginn kraftur**
a. handleggnum sem varð fyrir *meiri áhrifum* heilaslagsins?	5 ☐	4 ☐	3 ☐	2 ☐	1 ☐
b. gripi þeirrar handar sem varð fyrir *meiri áhrifum* heilaslagsins?	5 ☐	4 ☐	3 ☐	2 ☐	1 ☐
c. fótleggnum sem varð fyrir *meiri áhrifum* heilaslagsins?	5 ☐	4 ☐	3 ☐	2 ☐	1 ☐
d. fætinum/ökklanum sem varð fyrir *meiri áhrifum* heilaslagsins?	5 ☐	4 ☐	3 ☐	2 ☐	1 ☐

2. **Eftirfarandi spurningar eru um minni og hugsun.** ***Spurningarnar eiga við síðastliðna viku.***

**Hversu erfitt fannst þér að ...**	**Ekki erfitt**	**Svolítið erfitt**	**Miðlungs erfitt**	**Fremur erfitt**	**Afar erfitt**
a. muna það sem fólk var nýbúið að segja þér?	5 ☐	4 ☐	3 ☐	2 ☐	1 ☐
b. muna það sem gerðist daginn áður?	5 ☐	4 ☐	3 ☐	2 ☐	1 ☐
c. muna að leysa fyrirhuguð verkefni (t.d. að mæta á réttum tíma eða að taka lyfin)?	5 ☐	4 ☐	3 ☐	2 ☐	1 ☐
d. muna hvaða dagur vikunnar er?	5 ☐	4 ☐	3 ☐	2 ☐	1 ☐
e. einbeita þér?	5 ☐	4 ☐	3 ☐	2 ☐	1 ☐
f. hugsa hratt?	5 ☐	4 ☐	3 ☐	2 ☐	1 ☐
g. leysa dagleg vandamál?	5 ☐	4 ☐	3 ☐	2 ☐	1 ☐

3. **Eftirfarandi spurningar eru um líðan þína, breytingar á skapi þínu og getu þína til að stjórna tilfinningum þínum eftir heilaslagið.** ***Spurningarnar eiga við síðastliðna viku.***

Hversu oft ...	Aldrei	Einstökum sinnum	Stundum	Oft	Alltaf
a. fannst þér þú vera döpur/dapur?	5 ☐	4 ☐	3 ☐	2 ☐	1 ☐
b. fannst þér eins og enginn stæði þér nærri?	5 ☐	4 ☐	3 ☐	2 ☐	1 ☐
c. fannst þér þú vera byrði á öðrum?	5 ☐	4 ☐	3 ☐	2 ☐	1 ☐
d. fannst þér þú ekki hafa neitt til að hlakka til?	5 ☐	4 ☐	3 ☐	2 ☐	1 ☐
e. álasaðir þér þig fyrir mistök sem þú gerðir?	5 ☐	4 ☐	3 ☐	2 ☐	1 ☐
f. naustu lífsins eins og áður?	5 ☐	4 ☐	3 ☐	2 ☐	1 ☐
g. fannstu fyrir taugaspennu?	5 ☐	4 ☐	3 ☐	2 ☐	1 ☐
h. fannst þér lífið hafa tilgang?	5 ☐	4 ☐	3 ☐	2 ☐	1 ☐
i. brostir þú og hlóst a.m.k. einu sinni á dag?	5 ☐	4 ☐	3 ☐	2 ☐	1 ☐

4. **Eftirfarandi þættir eru um getu þína til að hafa samskipti við fólk og getu þína til að skilja það sem þú lest og heyrir í samtali.** ***Spurningarnar eiga við síðastliðna viku.***

Hversu erfitt fannst þér að ...	Ekki erfitt	Svolítið erfitt	Miðlungs erfitt	Fremur erfitt	Afar erfitt
a. segja nafn þess sem var auglitis til auglitis við þig?	5 ☐	4 ☐	3 ☐	2 ☐	1 ☐
b. skilja það sem sagt var við þig?	5 ☐	4 ☐	3 ☐	2 ☐	1 ☐
c. svara spurningum?	5 ☐	4 ☐	3 ☐	2 ☐	1 ☐
d. nefna hluti réttu nafni?	5 ☐	4 ☐	3 ☐	2 ☐	1 ☐
e. taka þátt í samræðum í hópi fólks?	5 ☐	4 ☐	3 ☐	2 ☐	1 ☐
f. eiga samtal í síma?	5 ☐	4 ☐	3 ☐	2 ☐	1 ☐
g. hringja í annan einstakling (þar með talið að finna rétt símanúmer og velja númerið)?	5 ☐	4 ☐	3 ☐	2 ☐	1 ☐

5. **Eftirfarandi spurningar varða athafnir sem þú framkvæmir mögulega á hverjum degi.** ***Spurningarnar eiga við <u>síðastliðnar tvær vikur.</u>***

**Hversu erfitt fannst þér að ...**	**Ekki erfitt**	**Svolítið erfitt**	**Miðlungs erfitt**	**Fremur erfitt**	**Gat alls ekki gert**
a. matast með hníf og gaffli (m.a. að skera matinn)?	5 ☐	4 ☐	3 ☐	2 ☐	1 ☐
b. klæða þig að ofan (frá mitti og upp úr)?	5 ☐	4 ☐	3 ☐	2 ☐	1 ☐
c. baða þig?	5 ☐	4 ☐	3 ☐	2 ☐	1 ☐
d. klippa táneglurnar?	5 ☐	4 ☐	3 ☐	2 ☐	1 ☐
e. komast tímanlega á klósett?	5 ☐	4 ☐	3 ☐	2 ☐	1 ☐
f. stjórna þvaglátum (slysalaust)?	5 ☐	4 ☐	3 ☐	2 ☐	1 ☐
g. stjórna hægðum (slysalaust)?	5 ☐	4 ☐	3 ☐	2 ☐	1 ☐
h. sinna léttum heimilisstörfum (t.d. þurrka af, búa um rúmið, fara út með ruslið eða vaska upp)?	5 ☐	4 ☐	3 ☐	2 ☐	1 ☐
i. fara í búðir?	5 ☐	4 ☐	3 ☐	2 ☐	1 ☐
j. sinna erfiðum heimilisstörfum (t.d. ryksuga, þvo þvott eða vinna í garðinum)?	5 ☐	4 ☐	3 ☐	2 ☐	1 ☐

6. **Eftirfarandi spurningar varða getu þína í að komast um heima hjá þér og úti í samfélaginu.** ***Spurningarnar eiga við <u>síðastliðnar tvær vikur.</u>***

**Hversu erfitt fannst þér að ...**	**Ekki erfitt**	**Svolítið erfitt**	**Miðlungs erfitt**	**Fremur erfitt**	**Gat alls ekki gert**
a. sitja án þess að missa jafnvægið?	5 ☐	4 ☐	3 ☐	2 ☐	1 ☐
b. standa án þess að missa jafnvægið?	5 ☐	4 ☐	3 ☐	2 ☐	1 ☐
c. ganga án þess að missa jafnvægið?	5 ☐	4 ☐	3 ☐	2 ☐	1 ☐
d. flytja þig úr rúmi yfir í stól?	5 ☐	4 ☐	3 ☐	2 ☐	1 ☐
e. ganga um í hverfinu (u.þ.b. 200 metra)?	5 ☐	4 ☐	3 ☐	2 ☐	1 ☐
f. ganga hratt?	5 ☐	4 ☐	3 ☐	2 ☐	1 ☐
g. ganga milli hæða upp einn stiga?	5 ☐	4 ☐	3 ☐	2 ☐	1 ☐
h. ganga upp nokkrar hæðir í stiga?	5 ☐	4 ☐	3 ☐	2 ☐	1 ☐
i. fara inn í og út úr bíl?	5 ☐	4 ☐	3 ☐	2 ☐	1 ☐

7. **Eftirfarandi spurningar eru um getu þína til að nota hendina sem hefur orðið meira fyrir áhrifum heilaslagsins.** ***Spurningarnar eiga við <u>síðastliðnar tvær vikur.</u>***

**Hversu erfitt fannst þér að nota hendina, sem varð fyrir meiri áhrifum heilaslagsins, þegar þú ...**	**Ekki erfitt**	**Svolítið erfitt**	**Miðlungs erfitt**	**Fremur erfitt**	**Gat alls ekki gert**
a. barst þunga hluti (t.d. fullan innkaupapoka með mat)?	5 ☐	4 ☐	3 ☐	2 ☐	1 ☐
b. snérir hurðarhúni?	5 ☐	4 ☐	3 ☐	2 ☐	1 ☐
c. opnaðir niðursuðudós eða krukku?	5 ☐	4 ☐	3 ☐	2 ☐	1 ☐
d. reimaðir skó?	5 ☐	4 ☐	3 ☐	2 ☐	1 ☐
e. tókst lítinn hlut upp af gólfinu (t.d. smámynt)?	5 ☐	4 ☐	3 ☐	2 ☐	1 ☐

8. **Eftirfarandi spurningar eru um hvernig heilaslagið hefur mögulega haft áhrif á getu þína til þátttöku í venjubundnum athöfnum, verkum sem eru þýðingarmikil fyrir þig og hjálpa þér í að finna tilgang með lífinu.** ***Spurningarnar eiga við <u>síðastliðnar fjórar vikur.</u>***

**Hversu oft hefur geta þín takmarkað þátttöku þína ...**	**Aldrei**	**Einstökum sinnum**	**Stundum**	**Oftast**	**Alltaf**
a. í vinnu þinni (launaðri vinnu, sjálfboðavinnu eða annarri vinnu?	5 ☐	4 ☐	3 ☐	2 ☐	1 ☐
b. í félagslegri virkni?	5 ☐	4 ☐	3 ☐	2 ☐	1 ☐
c. í þögulli afþreyingu (t.d. handavinnu eða lestri)?	5 ☐	4 ☐	3 ☐	2 ☐	1 ☐
d. í virkri/líkamlegri afþreyingu (t.d. íþróttum, útivist eða ferðalögum)?	5 ☐	4 ☐	3 ☐	2 ☐	1 ☐
e. í hlutverki þínu sem fjölskyldumeðlimur eða vinur?	5 ☐	4 ☐	3 ☐	2 ☐	1 ☐
f. í menningarviðburðum (t.d. að fara í leikhús eða á söfn) ?	5 ☐	4 ☐	3 ☐	2 ☐	1 ☐
g. við að stjórna lífi þínu eins og þú vilt?	5 ☐	4 ☐	3 ☐	2 ☐	1 ☐
h. við að hjálpa öðrum?	5 ☐	4 ☐	3 ☐	2 ☐	1 ☐

9. **Bati eftir heilaslag.**

Á kvarðanum 0 til 100, þar sem 100 stendur fyrir fullan bata og 0 stendur fyrir engan bata, hversu mikinn bata hefur þú fengið eftir heilaslagið?*Merktu svar þitt á kvarðann.*

______ 100 Fullur bati

—

______ 90

—

______ 80

—

______ 70

—

______ 60

—

______ 50

—

______ 40

—

______ 30

—

______ 20

—

______ 10

—

______ 0 Enginn bati
